# Research Progress of Near-Infrared Fluorescence Immunoassay

**DOI:** 10.3390/mi10060422

**Published:** 2019-06-24

**Authors:** Xiao-Hui Chang, Jie Zhang, Lin-Huan Wu, Yan-Kun Peng, Xiang-Ying Yang, Xiao-Lin Li, Ai-Jin Ma, Jun-Cai Ma, Guang-Quan Chen

**Affiliations:** 1 Beijing Inspection & Quarantine Testing Center, Beijing 100026, China; changxiaohui82@163.com (X.-H.C.); bjxchh@163.com (J.Z.); 2616yang@163.com (X.-Y.Y.); lixiaolin@163.com (X.-L.L.); chengq@163.com (G.-Q.C.); 2 Institute of Microbiology, Chinese Academy of Sciences, Beijing 100101, China; wulh@im.ac.cn (L.-H.W.); ma@im.ac.cn (J.-C.M.); 3 College of Engineering, China Agricultural University, Beijing 100083, China; ypeng@cau.edu.cn; 4 China National Institute of Standardization, Beijing 100191, China

**Keywords:** near-infrared fluorescence probes (NIFPs), immunochromatographic test strips (ICTSs), quantum dots, rare earth compounds, immunoassay, pathogen detection

## Abstract

Near-infrared fluorescence probes (NIFPs) have been widely used in immunoassay, bio-imaging and medical diagnosis. We review the basic principles of near-infrared fluorescence and near-infrared detection technology, and summarize structures, properties and characteristics of NIFPs (i.e., cyanines, xanthenes fluorescent dyes, phthalocyanines, porphyrin derivates, single-walled carbon nanotubes (SWCNTs), quantum dots and rare earth compounds). We next analyze applications of NIFPs in immunoassays, and prospect the application potential of lateral flow assay (LFA) in rapid detection of pathogens. At present, our team intends to establish a new platform that has highly sensitive NIFPs combined with portable and simple immunochromatographic test strips (ICTSs) for rapid detection of food-borne viruses. This will provide technical support for rapid detection on the port.

## 1. Introduction

Near-infrared fluorescent probes have a characteristic molecular structure with a highly conjugated polyene system. This exhibits a long emission wavelength over 650 nm up to 900 nm [1,2]. In this region, it has lower tissue autofluorescence, and less fluorescence extinction enhances deep tissue penetration with minimal background interference. Due to its high sensitivity and selectivity, fluorescence spectroscopy has a wide range of applications in analytical chemistry, especially in bioanalysis [3,4]. Most biomolecules have no fluorescence or weak fluorescence, and their detection sensitivity is low. In order to detect them with high sensitivity, people use fluorescent labeling reagents or fluoregenic reagents to label or derivatize the analytes. The formation of a covalent or non-covalently bound material with high fluorescence intensity greatly decreases the detection limit. Near infrared (NIR) fluorescence detection has obvious advantages in biological sample analysis.

**Figure 1:**
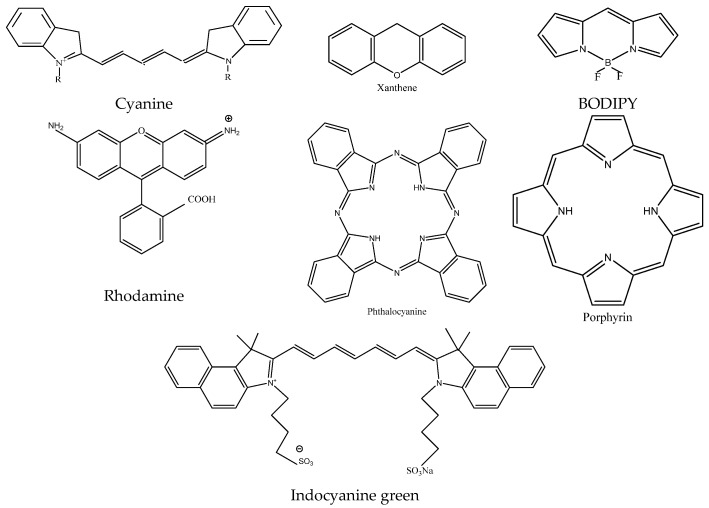
Basic chemical structure of near infrared fluorescent (NIRF) dyes.

Near-infrared fluorescent dyes are classified into the following categories, including cyanines, xanthenes fluorescent dyes, 4,4-difluoro-4-bora-3a,4a-diaza-s-indacene (BODIPYs), phthalocyanines, porphyrin derivates and other related fluorescent dyes (as shown in Figure 1). Many organisms and their tissues emit fluorescence under the excitation of visible light, which seriously interferes with the fluorescence detection of biological samples. For biological samples, the sample matrix and some impurities also have light absorption or fluorescence in this region (<600 nm). Light scattering from biological samples can cause more serious background interference, limiting the sensitivity of fluorescence analysis. Compared with conventional fluorescence (λem < 600 nm) detection, the light absorption or fluorescence intensity of the biological sample matrix is small in the near-infrared fluorescence (λem > 600 nm) region. Therefore, the background interference is greatly reduced, since the intensity of the scattered light is inversely proportional to the fourth power of the wavelength. As the wavelength increases, the Rayleigh scattering rapidly decreases, and the scattering interference is also greatly reduced.

In recent years, near-infrared fluorescent labeling reagents and detection techniques based on diode lasers with compact structure, good stability and low price have had higher sensitivity. The techniques have been used for near-infrared fluorescence immunoassays, flow cytometry, and fluorescence detection of biological active substances in high performance capillary electrophoresis separation. At the same time, with the continuous integration of various technologies, the development of many rapid detection devices such as laser fluorescence, sensors and immunodetection devices has promoted near-infrared fluorescent marker detection and analysis progress in the biological field [5]. 

NIR spectroscopy technology has many advantages in application areas. NIR spectroscopy can be used for food quality analysis, such as freshness of agricultural products, fruit firmness and quality, fruit sugar and acidity, the quality of baked products and detecting alcohol during alcohol fermentation in the food industry [6,7]. It also can detect changes in sugar content, prediction of different meat characteristics, determination of nutrients in dairy products and authentic identification of edible oil [8,9]. In agriculture, it can be applied in crop quality analysis and evaluation, crop variety resource identification and quality breeding and crop resistance index analysis [10,11]. In the field of pharmaceutical research, near-infrared spectroscopy can comprehensively analyze the grain size, crystal form, cleanliness, apparent density and optical rotation of the drug raw materials [12,13]. It can analyze pharmaceutical preparations as well as online monitoring and control of drugs [14]. Near-infrared spectroscopy is also widely used in other fields such as petrochemicals, polymer chemicals, basic organic chemicals and the textile industry [15,16]. Near-infrared spectroscopy has new developments in other fields such as microbiology and clinical medicine [17,18]. Currently, most of the research objects are bacteria and yeast and a few objects are fungi and algae [19,20,21].

## 2. Near-Infrared Fluorescent Probe Types

Near infrared fluorescence immunoassay is a novel lateral flow assay (LFA) that combines near-infrared fluorescence probes (NIFPs) with immunoassay. Near-infrared fluorescent probes have small background interference and strong tissue penetration. NIFPs have attracted more and more attention in recent years (As shown in Table 1). Due to the limited sensitivity of labeled probes based on color signals such as colloidal gold, enzymes and electrochemical signal generation probes are expensive and cumbersome to operate, and it is difficult to achieve one-step detection. NIFPs have become one of the most popular signal molecules. These probes are widely used in various bioanalytical fields [22]. NIFPs with emission spectra in the near-infrared region (wavelength 650–900 nm), which attracts attention in the field of analysis owing to their high signal-to-noise ratio and detection sensitivity. The basic chemical structures of near infrared fluorescent (NIRF) dyes are shown in Figure 1.

First, the organisms rarely self-fluoresce in the near-infrared region, so the analysis based on NIFP labeling is protected from background fluorescence. Second, the intensity of the scattered light is inversely proportional to the fourth power of the wavelength, so NIFPs with emitted light in the long-wave region are less affected by its interference. NIFPs have strong penetrability and small damage to biological tissues, which has been widely used in non-destructive testing and bio-imaging [22,23].

### 2.1. Near-Infrared Fluorescent Material

Although new forms of NIFPs are being synthesized, NIFPs mainly include traditional organic fluorescent dyes such as cyanine, rhodamine, and other thiazine dyes and oxazine dyes. They are widely developed as NIFPs, in which cyanine dyes are most favored for good biocompatibility. Cyanine dye-labeled biological samples mainly have non-covalent, covalent forms of electrostatic/hydrophobic interactions and bioconjugates with some biomolecules through reactive groups for labeling nucleic acids, proteins, immunoassays and bioimaging. Many studies have been focused on how to modify and redesign NIR probes to overcome their limitations.

#### 2.1.1. Cyanine

The cyanine dyes in the near-infrared region mainly include pentamethine and heptamethine cyanine dyes. Cyanine dyes display poor photostability and are prone to photooxidative bleaching. The photooxidation of cyanine dyes has the following characteristics such as the photooxidation reaction in solution conforms to the first-order reaction kinetics; when the structure of the methine chain in the cyanine dye is the same, the substitution of heteroatoms on the heteronuclear core increases, and the stability decreases (indole > oxazole > thiazole > selenazole). When the heteronuclear core structure is the same, the longer the hemi-chain, the worse the photostability. That is very important for the design of near-infrared cyanine dyes with good light stability. Cyanine dye-labeled biological samples mainly have non-covalent, covalent forms of electrostatic/hydrophobic interactions and bioconjugates with some biomolecules through reactive groups for labeling nucleic acids, proteins, immunoassays, and bio-imaging [24]. Among them, thiazole orange, oxazole orange dimer and monomer are mainly used for DNA analysis. These fluorescent probes have weak fluorescence, and the fluorescence is markedly enhanced after labeling DNA. The cyanine dyes feature good optical properties such as high absorption and long absorption/emission wavelength. Hemi-cyanine dyes and their analogues have been used for NIRF platforms in in vivo imaging [25].

There is less literature on the direct detection of non-covalently labeled proteins with near-infrared cyanine dyes. It has been reported that direct measurement of protein by fluorescence detection may result in higher sensitivity than hemimethine cyanine absorption spectrophotometry [26] (as shown in Table 2). In the presence of protein polyglutamate, some researchers measured the fluorescence of the protein. The detection limits of bovine serum albumin (BSA), Human Serum Albumin (HAS) and C-IgG were 37, 40, 43 ng/mL, respectively [26]. These methods were applied to serum samples and researchers had found that the test results were satisfactory. Some solvent-sensitive merocyanines are also covalently bound to proteins for studying changes in protein conformation in living cells. Capillary electrophoresis was used as a separation method to detect the fluorescence and electrophoretic behavior of non-covalent binding of squaraine NN127(III), NN525(IV) and SQ-3(V) to proteins [27]. Fluorescence detection in the near-infrared region is more suitable for bio-tissue analysis than in the visible region. Light penetration into tissue is the basis for optical imaging of living tissue. The depth of this light penetration is closely related to the wavelength of light. With the wavelength of light greater than 600 nm, the depth of the light-permeable tissue can reach several centimeters, so that a relatively large volume of tissue can be imaged for disease diagnosis. The most widely used in biological tissue imaging is indocyanine green (XII), which is mostly used for fluoroscopy of clinical living tissues. In recent years, it has been tested as an animal and human cancer tissue. Some other cyanines are also used for the detection of cancer in vivo.

At present, many cyanines ave been commercialized. For examples, Hirayama and his colleagues have combined a NIR cyanine dye with a sulfur-rich receptor [28]. This modle show a selective and sensitive turn-on response to Cu^+^ in living cell. Lee et al. further improved and derivatized it by DOFLA (diversity-oriented fluorescence library approach) [29]. They obtained more than 40 NIFPs with excellent physicochemical properties. The light stability of AZA396 was 60 times higher than that of Bodipy Fl. Researchers are constantly working on the synthesis of novel near-infrared organic probes, as well as improvements in key traits such as water solubility, quantum yield, chemical and photostability, and biocompatibility of traditional near-infrared fluorescent dyes [30]. Aggregation of dyes or dye combinations can cause severe fluorescence quenching and many studies have focused on improving the water solubility of dyes [31]. Since it was first discovered and reported by Waggoner et al. in 1993, the attachment of sulfonate groups to the aromatic ring has been effective in increasing the water solubility of NIFPs [32]. This conclusion was also confirmed by Cheng et al. [33]. The well-known commercialized near-infrared organic molecules Cy5.5 and Cy7 from Amersham Biosciences are all sulfonate phthalocyanine dye structures. Its extension of the nitrogen-containing heterocycles leads to a red shift by 20 nm. The elongation of the middle polymethine by the vinylene bond (CH=CH) results in a red shift by 100 nm [34].

In order to effectively increase the fluorescence intensity of dyes to determine trace target analytes, researchers continue to explore efficient signal amplification strategies. For example, a large amount of fluorescent dye is wrapped in nanoparticles to form a near-infrared nanoparticle probe with higher fluorescence intensity. This has been widely confirmed to effectively improve the detection signal intensity while improving the chemical and photostability of the labeled molecule [35]. Furthermore, based on the surface plasmon resonance of the metal nanostructure, the fluorescence intensity of the near-infrared fluorescent dye can also be significantly improved. As studies have shown, by using rough metal surfaces such as silver island films or gold nano-shells, the signal intensity of phthalocyanine green can be increased by 20 and 50 times respectively [36,37]. In addition, coating the nano-microspheres with a multi-polymer material coated with a near-infrared fluorescent dye has been indicated to improve the biocompatibility of the dye. Kim et al. have improved the compatibility of dyes with cells by encapsulating Cy5.5 in a hydrophilic polymer. NIFP could be used to monitor the imaging changes of cell structure in early stage of apoptosis [38].

#### 2.1.2. Near-Infrared Fluorescent Dye Containing Tetrapyrrole-Based Groups

Tetrapyrrole group-containing dyes such as porphyrins and phthalocyanines are very important class of NIFPs. Scholars have applied porphyrin probes to the structure, properties and functions of biological macromolecules such as DNA and proteins [39]. Small porphyrin molecules act on them and their properties and structural changes are determined by spectroscopic techniques such as fluorescence and phosphorescence. The fluorescence spectrum of phthalocyanine is located at 600–700 nm. Compared with porphyrin, phthalocyanine has good stability to light, oxygen and heat. Water-soluble phthalocyanines are increasingly used in biochemical analysis for their unique absorption and fluorescence properties. 

Chen et al. used tetraamine aluminum phthalocyanine as the red region fluorescent substrate to establish a fluorescence analysis method for the determination of peroxidase and hydrogen peroxide [40]. Some research group used tetrasulfonate aluminum phthalocyanine as a fluorescent probe to establish a method for the continuous determination of serum protein, albumin and globulin. They studied interaction between phthalocyanine and bovine serum albumin [40]. Some researchers have combined the fluorescent probes of tetrapyrrole with some biomolecules to form bioconjugates in the diagnosis of tissue bioimaging. These biomolecules include antibodies, antibody fragments, peptides, serum proteins and estrogens to improve selectivity. These derivatives have great potential in the treatment of cancer. Karunakaran et al. have developed a hydrophilic porphyrin (THPP) and its derivative (Zn-THPP). They displayed superior quantum yield and excellent free radical generation rates [41]. In comparison with the clinical drug Photofrin, THPP exhibited higher photodynamic activity. Moreover, THPP rapidly permeated into cells and localized in the nucleus, demonstrating its potential application as a NIR probe for PDT as well as nucleus imaging. The low solubility and big size of phthalocyanine dyes affect other properties of biomolecules, which limits their wider application in bioanalysis.

#### 2.1.3. Xanthene Fluorescent Dye

Xanthene fluorescent dyes mainly include two classical fluorescent dyes, fluorescein and rhodamine. Naphthalene ring fluorescein is in the near infrared spectral region. Some near-infrared rhodamine fluorescent dyes such as Rhodamine 800 (IX) and Texas Red (X) are used in bioanalysis [42]. Rhodamine 800 has performed fluorescence polarization tracing on chicken tissue and has also been used to study mouse mitochondrial membrane potential. However, these fluorescent probes from classical rhodamine dyes can only emit visible light at wavelengths of 500–600 nm, and these probes can not be used in biological imaging. Their fluorescent properties can be altered by a ring open/close process or photo-induced electron transfer (PET) [43,44]. These Rhodamine dyes have great molar extinction coefficients and resistance in photobleaching such as PET, oxidation-reduction, and spiro ring opening of xanthenes [34]. Texas Red also has some applications in bioanalysis. Molecular Probes Inc. of the United States has introduced a series of Alex Fluoro’s xanthene-like fluorescent probes with wavelengths ranging from the visible region to the near-infrared region [45]. Because these Alex Fluoro fluorescent probes contain a sulfonic acid group, they are water soluble and suitable for labeling and analysis of biological samples. In addition, the introduction of a sulfonic acid group increases the polarity of the molecule and reduces its aggregation in water.

A small Stokes’ displacement may result in back scattering from biological samples and self-quenching. Gong and colleagues had expend a p-conjugated system of xanthene to develop an efficient NIR fluorophore [46]. The fluorphore has a large Stokes’ displacement and high fluorescence quantum yield.

#### 2.1.4. Thiazine and Oxazine Near-Infrared Fluorescent Dyes

Both thiazine dyes and oxazine dyes contain amino groups which can be used for labeling. Currently, these dyes are mainly Nile Blue (XI), Nile Red, Methylene Blue (XII) and Oxazine 750. Nile Blue (XI) derivative is used for HPLC-LIF analysis of carboxylic acid, the detection limit can reach 3.98 × 10^−11^ mol/L, the detection limit of aromatic acid in plasma is 7.33 × 10^−11^ mol/L [47]. The thiazide and oxazine-based near-infrared fluorescent probes have advantages of easier synthesis and smaller molecules, but their fluorescence quantum yield is low, which limits their application.

#### 2.1.5. Boron Difluoride-Dipyrromethane (BODIPY) Class

The boron difluoride-dipyrromethane (BODIPY) fluorescent dye has a high molar absorptivity (E > 80,000 L·moL^−1^·cm^−1^), a high fluorescence quantum yield. Its fluorescence is not sensitive to the polarity of the solvent, pH, narrow fluorescence peaks, long fluorescence lifetime and good light stability. A styryl group was introduced at the 3 position of the dye precursor, and two phenyl groups (XV) were introduced at the 1st and 3rd positions to synthesize two long-wavelength fluorescent dyes having a wavelength exceeding 600 nm [48]. The ortho-benzene dicarbonyl compound is used as a raw material, and reacts with hydroxylamine to form an isodecadiene compound, which is then complexed with boron trifluoride to form a BODIPY of a large conjugated system [49]. The maximum emission wavelength of such fluorescent dyes exceeds even 700 nm. In 2005, some BODIPY containing different active functional groups have also been synthesized, such as active esters and carboxylic acids [50]. 

Currently, some studies adopt two main strategies to shift BODIPY into NIRF dyes, modify the phenyl rings and merge the 3- and 5-phenyl rings with the aza-BODIPY core, which forms six-membered rings and reduces the torsion angles formed by peripheral phenyl groups and the central core [49,51]. Enhancement of co-planarity between phenyls and the central core results in NIR batho-chromic shifts, probably due to electron delocalization. Yang’s group has developed bromo-substituted BODIPY-containing thienopyrrole moieties. They found exposure of these products in the NIR region exhibited a high singlet oxygen quantum yield that leads to photo cytotoxicity [52].

### 2.2. Near-Infrared Fluorescent Quantum Dot

Quantum dots (QDs) known as semiconductor nano-microcrystals, have been widely used in bioanalysis and medical diagnostics recently as a new class of fluorescent probes due to their excellent optical properties [53]. The fluorescence emission spectrum of this probe with adjustable particle size and composition ensures its feasibility as a near-infrared labeled probe. Near-infrared quantum dots refer to quantum dots with emission wavelengths between 650 and 900 nm, and have the dual characteristics of near-infrared and quantum dots. Compared with traditional organic fluorescent dyes, QDs exhibit high quantum yield, large Stokes’ shifts and can endure photobleaching [54]. As an emerging biological probe, applications of quantum dots are still expanding in scope. However, this cannot replace the traditional organic small molecule fluorescent probe, and can only be used as a powerful supplement to the existing organic small molecule fluorescent probe. 

However, biocompatibility remains to be further explored because of its potential toxicity to living tissue. In order to solve the potential acute and chronic toxicity of NIR quantum dots, a less toxic coating is often considered in the design of QDs [55,56]. Currently, quantum dot-based chromatographic test strips have been widely used in food safety, environmental monitoring, medical diagnosis and other fields [57,58,59,60]. However, studies on the preparation of dual-function quantum dot (QD) fluorescent probes with dual-targeting action are rare. Cui et al. prepared a dual-targeting CdTe/CdS QD fluorescent probe with a bovine serum albumin–glycyrrhetinic acid conjugate and arginine-glycine-aspartic acid successfully [61]. This probe could induce the apoptosis of liver cancer cells and showed enhanced targeting in vitro cell imaging.

### 2.3. Near-Infrared Fluorescent Rare Earth Complex

Complexes of rare earth elements (lanthanides) containing Nd^3+^, Er^3+^, Yb^3+^ and Tm^3+^ in the near-infrared region have been widely developed in recent years [62,63,64,65]. Compared to NIFPs such as organic dyes and semiconductor nanocrystals, Near-infrared fluorescent rare earth complexes have unique advantages such as large Stokes’ displacement, long fluorescence lifetime and no photobleaching [66]. Due to the low extinction coefficient, the use of free lanthanides is often hindered. Therefore, researchers need a photon converter to process the vibrational overtone spectrum induced by -OH, -NH and –CH [67]. In order to overcome the difficulties, many researchers are committed to the further optimization of NIF lanthanides. For example, Foucault-Collet et al. developed unique NIF rare earth metal-organic frameworks (MOFs) that encapsulate a large number of NIF-emitting Yb^3+^ ions with the sensitizer phenylenevinylene dicarboxylate (PVDC) in a small volume [68]. This structure not only provides a new method for sensitization and protection of lanthanides, but also greatly improves the detection sensitivity due to the increase in the number of probes carried per unit volume. In addition, incorporation of rare earth elements into laser materials or nanocrystals has also been shown to effectively improve their optical properties [69,70].

The rare earth complex luminescent probe mainly comprises a complex fluorescent probe of Eu^3+^ and Tb^3+^. Its Stoke’s displacement is large and the fluorescence lifetime can reach milliseconds, so it is widely used in biotechnology and time-resolved fluorescence detection technology. The main emission peak of Tb^3+^ is around 550 nm, while the main emission peak of Eu^3+^ is around 620 nm. Rare earth complex fluorescent probes are sensitive to external environmental pH and coexisting ions such as alkali metal ions and halogen ions. Therefore, they are often used in chemical and biological sensors. The detection limit of lecithin was determined by direct fluorescence spectrophotometry with Eu^3+^-tetracycline complex to reach 3.9 × 10^−8^ mol·L^−1^, and it was successfully used for the determination of lecithin in serum samples [71]. This complex is also used to detect nicotine dinucleotide adenine. The sensitivity of the Eu^3+^- oxytetracycline complex for detection of adenosine triphosphate can reach 2.67 × 10^−9^ mol·L^−1^ [72]. The complex of Eu^3+^ is also used for sensitive detection of nucleic acids. In the system of Eu^3+^-benzoylacetone-hexadecy lammonium bromide, the presence of nucleic acid enhances fluorescence, so this system is used to detect the sperm DNA of black carp. The detection limits of calf thymus DNA and yeast RNA were 0.33, 0.21 and 0.99 ng·mL^−1^ respectively [73]. Rare earth complex fluorescent probes have also been reported for cancer detection in vivo [74]. A Eu^3+^ complex is also synthesized and used for staining and labeling of the nucleus and exhibits highly selective labeling of the nucleus.

### 2.4. Single-Walled Carbon Nanotubes

As a new type of carbon material, single-walled carbon nanotubes (SWCNTs) have great potential for application in the biomedical field due to their special nanostructures and excellent optical, mechanical, electrical and magnetic properties [75]. More and more researchers are beginning to pay attention to SWCNTs [70]. SWCNTs are photoluminescence with an emission spectrum greater than 1000 nm. It is an ideal near-infrared fluorescent material. Compared with other probe molecules, it has the following advantages. First, because SWCNTs have strong emissions in the near-infrared region of 1000–1400 nm and the Stokes’ displacement is large, their interference with autofluorescence is significantly reduced compared with other fluorescent probes [76]. Secondly, SWCNTs are extremely short. Fluorescence lifetime (t < 2 ns) can effectively eliminate non-radiative deactivation, which makes the fluorescent probes have high fluorescence quantum yield. In addition, the fluorescence emitted by SWCNTs is highly resistant to photobleaching and has good stability.

Due to its high quantum yield, small background interference, and good light stability, SWCNTs have been widely used in recent years as ideal NIFPs for in vivo and in vitro bio-imaging, and immunoassays for important diagnostic and therapeutic marker molecules [77,78].

## 3. Applications of Near-Infrared Fluorescence Immunoassay

### 3.1. Antibiotic Test

In 2016, Chen et al. developed a multiple lateral flow immunoassay based on NIR by combining near-infrared labeling with broad-spectrum-specific monoclonal antibody/receptor as a detection complex. The method could simultaneously detect four antibiotic families in milk such as β-lactams, tetracyclines, quinolones and sulfonamides within 20 min [79].

### 3.2. Medical Diagnostic Marker Molecular Detection

Compared with a few NIR immunoassays for antibiotic tests, this method is more widely used in immunoassays of important marker molecules in diagnostics. So far, NIFPs have been successfully developed for immunotiter plates, fiber-optic immunosensors, and capillary blotting, capillary electrophoresis immunoassays and immunochromatographic assays [80,81,82]. A variety of different immunoassay models such as strips are used to detect key proteins for medical diagnosis [83].

In the early 1990s, NIFPs were first applied in immunoassays. Researchers achieved quantitative determination of human immunoglobulin by adding an excess of NIR-labeled antibody and subsequent fluorescence detection in an antigen-coated polyethylene microtiter plate [84]. Daneshvar et al. designed and developed a fluorescent fiber-optic immunosenseor (FFOI) for near-infrared labeling of human IgG [80]. An antibody is immobilized on the sensing end of FFOI for the identification and capture of trace specific antigens. The immune mode is sandwich type, which can be completed within 10–15 min with a detection limit of 10 ng/mL. In a follow-up study, the Dye1 used in the above studies was replaced by a water-soluble NIR dye. The FFOI system was further confirmed to efficiently quantify human IgG and effectively sensitize one order of magnitude, while FFOI can also be used in the detection of *Legionella pneumophila* [85]. The detection sensitivity of the FFOI system is comparable to that of enzyme-linked immunosorbent assay (ELISA), and it has many advantages such as short operative time, low detection cost and suitable for on-site detection. It was found that HITCI and BSA combined with enhanced fluorescence. HITCI (VI) non-covalently binds to bovine serum albumin BSA, and its fluorescence properties and labeling behavior were detected by CE-LIF (Capillary electrophoresis-laser-induced fluorescence).

Weissleder et al. used a biological conjugate of active protease-near-infrared cyanine to detect cancer. Many cancers overexpress some small peptide receptors (Receptors), such as Somatostain [86] Folate receptors are also specifically expressed by many cancers, so cyanine-folate conjugates are also used for detection of cancer. The cyanine dye can be detected by binding a reactive reactive group to an antibody or antigen protein to form a dye-protein conjugate. In 1997, Williams et al. attempted a near-infrared fluorescence immunoassay on a nitrocellulose membrane for the first time, opening the first step in the use of near-infrared fluorescent probes for solid-phase immunoassays [87]. Williams and Peralta used heptamethine cyanine dyes for solid-phase immunoassays to measure trace amounts of human immunoglobulin. Immunological reactions were performed with fluorescent probe-labeled GAHG (goat anti-human immunoglobulins) and human immunoglobulin. Because the hydroxyl group produced by the peroxide from hydrogen peroxide quenches the fluorescence, it can be used to detect the activity of the enzyme. CEIA (capillary electrophoresis-based immunoassay) is an analytical method for separation and detection by capillary electrophoresis using the differences in electrophoretic behavior between antigen-antibody complexes and free antigens and antibodies. This effectively simplifies the detection procedure, but its practical application is still hindered by the large interference of the scattered light emitted by the membrane matrix, the non-specific binding of the membrane, and the difficulty of matching with the microtiter plate immunoassay. In a subsequent study, the aforementioned heptamethine cyanine dye NN382 was effectively reduced by using a water-soluble, negatively charged sulfonate group to effectively reduce the non-specific binding of the same negatively charged membrane matrix to the dye-antibody complex. The trouble has been effectively solved and the development of solid phase NIFIAs has been promoted. Since then, Zhao et al. developed a solid-phase near-infrared immunofluorescence assay called capillary imprinting, which can be used for direct detection of peptides in complex biological fluid matrices [88]. This method increases the detection sensitivity of the target analyte dynorphin by a factor of 1000 compared to commercial hybridization plates.

In addition, Silva et al. developed an optical immunosensor based on the near-infrared dye Cy5 for the determination of disease infection in sheep *Brucella* sp., which can achieve a *Brucella* sp. antibody in serum of sick sheep (0.005–0.11 mg/mL) quantitative analysis [89]. According to the difference in electrophoretic behavior between antigen-antibody complexes and free antigens and antibodies, Cy5 was also used for a capillary electrophoresis immunoassay of IgA secreted in human saliva. The Cy5 labeling assay for immunoglobulin A secreted in human saliva has also been reported. The separation of antigen-antibody complexes and free antigens is best using a boric acid buffer solution containing 150 mmol·L^−1^ boric acid and 1% CHAPS PH 8.5. It was used for normal human and patient saliva sample determination. MeCy5-OSu(VII) is a newly synthesized cyanine dye near-infrared fluorescent labeling reagent. With biopolyamine as the analysis object, a new method for capillary biophoresis/laser induced fluorescence detection of biopolyamines was established. The linear range is 10–200 nmol·L^−1^, the detection limit is 0.8–3.0 nmol·L^−1^. This method has been used for the determination of biopolyamines in red blood cells of normal human and bladder cancer patients.

In recent years, novel NIFPs other than organic fluorescent dyes have also been introduced into near-infrared fluorescent immunoassay systems. For example, Deng et al. prepared a novel core/shell NIF nanoparticle by encapsulating the inexpensive near-infrared fluorescent dye methylene blue in a hydrophobic silica gel shell [90]. The immunoglobulin was used to determine the alpha fetus in the whole blood sample. protein. This special structure exhibits higher fluorescence intensity and better stability than conventional dye-coated silicon nanoparticles, thereby preventing interference from dye leakage and exogenous quenching factors. In addition, dual stabilizer-modified CdTe, CdTe/CdS core (thin)/shell (thick) and CdTe and CdSeTe/CdS/ZnS with mercaptopropionic acid as stabilizers quantum dot near-infrared electrochemiluminescence immunosensors have also been developed for the detection of fetal protein antigens, human IgG and carcinoembryonic antigens respectively [91,92,93]. The above system utilizes a near-infrared fluorescence resonance energy transfer system to measure the distance effect caused by the immune reaction between a near-infrared quantum dot-labeled protein and another probe (such as gold particles), and the fluorescence is caused by the induced energy transfer. The change in intensity enables high-sensitivity quantitative detection of target analytes. In addition to NIR-QDs, the emerging NIR fluorescent material SWCNTs (single-walled carbon nanotubes) have also been used in IgG immunoassays by Iizumi et al. [78]. By detecting co-immunoprecipitation between IgG-bound SWCNTs and immunomagnetic beads linked to protein G, the system can measure target analytes at concentrations as low as 600 pmol/L.

The lanthanide ion represented by lanthanum can emit fluorescence under specific excitation light, and has been developed into a novel fluorescent labeling probe for ICTSs in recent years. Because of its unique emission half-life longer than other fluorescent probes and background materials, lanthanides have been well developed as powerful labeling probes for time-resolved immunofluorometric assays. By delaying the measurement time, after all short-lived background fluorescence and excitation photons are completely decayed and then measuring the fluorescence signal of the lanthanide ions, the background interference can be effectively reduced or even eliminated, thereby improving the detection sensitivity by at least two orders of magnitude. According to Xia et al. and Zhang et al., the detection limits of ICTSs labeled with ruthenium nanoparticles are up to 100-fold relative to colloidal gold-labeled chromatographic strips and ELISA [94,95]. In addition, the marker has a large Stokes’ displacement, which can effectively distinguish the excitation spectrum and the long-wavelength emission spectrum, which not only reduces the requirements on the filter system, but also effectively eliminates background fluorescence interference [96]. Fluorescence of many different colors can be obtained by changing the species of lanthanide ions, making the probe suitable for multivariate detection. For example, on the surface of silica nanoparticles, Eu^3+^ and Tb^3+^ were covalently labeled for double labeling. Xu et al successfully detected the simultaneous detection of hepatitis B virus surface antigen and e antigen. In addition, the lanthanide ion-coated nanoparticles prepared in this study are highly stable and can be stored for up to two years [97].

One of the early pathological markers of Alzheimer’s disease (AD) is the deposition of amyloid-beta (Aβ) plaques in the brain. Optical imaging and particularly NIRF imaging has become a safe, low-cost, real-time and widely available technique that provides an attractive method for in vivo detection of Aβ plaques in many different imaging techniques. In 2015, Tong et al. briefly outlined the latest developments in NIRF Aβ probes and their applications in vitro and in vivo [98].

Researchers synthesized a specific COX-2 probe celecoxib-MPA probe (CMP), based on celecoxib and a water-soluble near-infrared dye dye ICG-Der-02 (MPA) for monitoring COX-2. The results suggested that the probe CMP, with favorable hydrophilic property, good biocompatibility, long-term observation, excellent targeting ability and optical imaging capability, and it could serve as a promising probe for real-time monitoring of COX-2 in inflammation and tumor [99].

Despite their high sensitivity, NIFPs in immunoassays are still lacking in simplicity, so its user friendliness needs further improvement. To overcome this shortcoming, Swanson and D Andrea developed quantitative immunochromatographic test strips (ICTSs) based on NIFPs for single and multiple-synchronous detection of interleukin-6 and C-reactive protein. In 2013, Swanson developed a quantitative ICTSs based on a near-infrared fluorescent probe. The NIR dye was coupled to the selected antibody and integrated into the LFA. The test strip can detect single and multiple simultaneous detection of interleukin-6 and C-reactive protein [83]. The high signal-to-noise ratio of NIFPs makes the detection limit of the test strip as low as pg/mL. That is equivalent to ELISA. In summary, NIR-labeled ICTSs provide a powerful tool for the evaluation of biomarker proteins in a real-time assay environment.

### 3.3. Applications in Microorganisms

Although current in vitro and in vivo bio-imaging is still the main application field of NIFPs, its application in immunoassays has not stopped over many years since Boyer first researched it in 1992 [84]. With continuous exploration of new NIFPs and continuous development of immunoassay technology, the combination of both has become increasingly popular in many analytical fields. LFA is the most powerful immunoassay for immediate testing due to its simple operation and portability. The application of NIFPs in LFA will undoubtedly provide a valuable platform on-site, high-sensitivity bioanalysis in future.

In 2013, Cheng et al. extracted the anti-pulmonary Legionella LP antigen to prepare immunofluorescent LP antibody kit, and explored application value of near-infrared fluorescence detection of *Legionella pneumophila* (LP) antigen [100]. It was found that this method is not related to other common bacteria. Cross-reactivity occurs with a minimum of 10 ng/ml, with good stability and repeatability. In recent years, some scholars have combined near-infrared technology with the immunomagnetic bead coupling method for quantitative detection. Zhou et al. labeled the monoclonal antibody targeting larabinomannan (LAM) with a near-infrared fluorescent dye, and the LAM method for detecting *Mycobacterium tuberculosis* by targeting the multi-antibody of LAM coated on the surface of the nanomagnetic beads [101]. They used a double antibody sandwich method to magnetically separate the conjugate and the free substance, and then used a portable near-infrared fluorescence detector to detect the fluorescence intensity of the magnetic conjugate, thereby detecting the LAM content in the sample to be tested, and found that the minimum detection limit of the method was 0.5 ng/mL. In 2018, Lin Chen et al. developed a new LFA based on NIR fluorescent dyes to detect anti-dengue virus (DENV1) IgG antibodies. The results of NIR-LFA were compared to those of Panbio Dengue IgG ELISA and the Dengue Duo IgM/IgG Kit. They identified 19 confirmed DENV1 positive samples by NIR-LFA with 95% sensitivity [102].

### 3.4. Our Team’s Research on Near-Infrared Fluorescence Immunoassay

Foodborne pathogens are one of the most important threat factors for food poisoning incidents worldwide. However, traditional microbial culture-based assays are time-consuming and labor-intensive, failing to provide timely data to effectively reduce the incidence of foodborne illness. Therefore, whether it is from the control of product quality by food companies, or the government’s effective supervision of food safety, so as to protect public health, we urgently need a faster and independent method for detecting food-borne pathogenic microorganisms. Although current colloidal gold-based LFA are still the gold standard for rapid detection of pathogens, the labeling technique is still limited by its low sensitivity and inability to accurately quantify defects. Fluorescent probes are methods that use optical properties of fluorescent molecules to study some of the physical, chemical, and physical properties of a particular environmental material at the molecular level. It has high sensitivity and wide dynamic response range, so it is widely used in biological macromolecules. In the near-infrared region, biomolecules have weak self-fluorescence and small background interference, and high sensitivity is obtained in this region. Therefore, the research of NIFPs has become a research hotspot in recent years, showing great potential in biological analysis.

At present, our team has developed an approach for detecting pathogens such as *Salmonella, Vibrio parahaemolyticus, Vibrio cholerae* and *Listeria monocytogenes* by near-infrared immunoassay (as shown in Table 3). In 2017, we used near infrared fluorescent marker of *Vibrio parahaemolyticus* monoclonal antibody, *Vibrio parahaemolyticus* polyclonal antibody and goat anti mouse IgG polyclonal antibody was coated on nitrocellulose membrane as the detection line and the control line. We have developed the detection of *Vibrio parahaemolyticus* near infrared later flow assay strips and supported the standard substance. The results showed that the near infrared spectroscopy technique has good specificity and high sensitivity for *Vibrio parahaemolyticus*, the lowest detection limit is 1.2 × 10^2^ CFU/mL. There was no cross-reaction with *Salmonella*, *Staphylococcus aureus*, *Escherichia coli* and *Listeria monocytogenes*. Compared with the traditional detection method, the detection time of near infrared fluorescence method is the shortest, and the detection limit is close to that of RT-PCR .his near infrared immunochromatographic method is ompleted in 45 min. It could be used for efficient detection of *Vibrio parahaemolyticus* in food and provide reliable technical support for food safety supervision.

## 4. Conclusions and Prospects

At present, near-infrared spectroscopy technology has developed with the development of different fields such as computer science, chemometrics, photomaterial science and measuring instruments, and has become a widely used analytical tool to solve some difficulties in quick detection challenges. This technology requires qualitative and quantitative analysis of unknown samples by establishing a calibration model. Because the absorption spectrum is a superposition of the absorption spectra of the contained compounds, and the map has a certain similarity, the map is complicated and difficult to resolve. The further popularization of near-infrared spectroscopy in the field of detection will be a great challenge. Therefore, near-infrared spectroscopy technology needs to be combined with characteristics of actual detection to develop near-infrared spectroscopy special information for each system and the processing technology of other disciplines, so that it can be better applied. With the development of NIR analysis technology and other fields of technology and the continuous expansion of its application, it will play an important role in the modernization of analytical tools. 

Since labeled molecules play a decisive role in the development of immunochromatography technology platforms, it is particularly important to develop ideal probes with high quantum yield, good stability, small background interference and easy labeling. The existing probe development mostly relies on simplicity to obtain high sensitivity and accuracy, which to some extent undermines the superiority of ICTSs in the field and in real-time detection. Therefore, more innovative research should be devoted to the development of highly accurate probes. At present the near-infrared fluorescent probe has excellent potential, but it is mainly used for biological imaging research, and the application on the ICTSs is almost blank. Previous studies have shown that near-infrared fluorescent probe labels are 100 times more effective than traditional colloidal gold test strips in detecting human immuno-deficiency virus (HIV).

**Figure 2:**
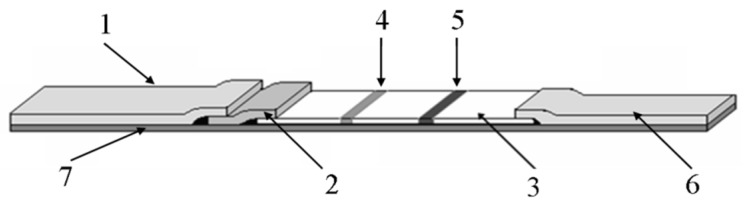
The structure of low-noise excitation fluorescence detection of *Vibrio Parahemolyticus* immunochromatographic test dipstick. (1: sample pad; 2: conjugate pad; 3: antibody carrying membrane; 4: test line; 5: control line; 6: absorbent pad; 7: Bottom plate.).

In recent years, our team has developed a method for detecting pathogens including *Salmonella*, *Vibrio parahaemolyticus*, *Vibrio cholerae* and *Listeria monocytogenes* by near-infrared immunoassay (as shown in Table 3). Our test strip is made up of five parts, including a Bottom plate, a sample pad, a conjugate pad, an antibody carrying membrane and an absorbent pad. As shown in Figure 2, the sample pad, conjugate pad, antibody-carrying membrane and absorbent pad were located in turn along the length direction of the Bottom plate, and overlap each other. The antibody-carrying membrane is located in the center of the base plate, which is provided with spaced apart test and control lines, test lines close to the bond pads, and control lines near the absorbent pads. The conjugate pad is sprayed by rat anti-*Vibrio Parahemolyticus* monoclonal antibody, test line marks rat anti-*Vibrio Parahemolyticus* polyclonal antibody, and control line marks Goat anti-mouse IgG polyclonal antibody. The emission wavelength of low noise excitation fluorescence dye is 650–1000nm. A kind of fluorescent molecules DyLight800 which excitation and emission wavelength are 777 and 790 nm is used. The control lines and test lines are arranged in parallel, and the line spacing between test lines and control lines is 0.5 cm.

Our team is working on foodborne viruses such as rotavirus, norovirus and hepatitis A virus, and we could develop near-infrared ICTSs using NIFPs-labeled antibodies to detect these foodborne viruses. Our team intends to establish a new platform which has a highly sensitive near infrared probe combined with portable and simple ICTSs for rapid detection of food borne viruses. This will provide technical support for a rapid detection on the port.

## Figures and Tables

**Table 1:** Types and characteristics of near-infrared fluorescent probes.

NIFPs Types	NIFPs (Near-Infrared Fluorescence Probes)	NIFPs Advantages	Disadvantage	Reference
Near-infrared fluorescent material	AZA396	Higher stability than BodipyF1	-	[29]
	Cy5.5, Cy7	Water solubility increased	Poor photostability	[33]
	Near-infrared nanoparticle probe	Higher signal intensity, chemical and photostability	-	[35]
	Silver island films resonance	Signal intensity increased	Poor biocompatibility	[36]
	Gold nano-shells resonance	Signal intensity increased	Poor biocompatibility	[37]
	Nano-microspheres with a multi-polymer material p-conjugated system of xanthenes Rhodamine	Improve the biocompatibility A large stokes’ displacement Great molar extinction and resistance in photobleaching	Self-quenching and back scattering	[38] [46] [52]
Near-infrared fluorescent quantum dot	Semiconductor nano-microcrystals	High quantum yield, strong anti-photobleaching ability and Concentrated emission spectrum	Its potential toxicity to living tissue	[53]
Near-infrared fluorescent rare earth complex	Lanthanides containing Nd^3+^, Er^3+^, Yb^3+^ and Tm^3+^	Large Stoke’s displacement; Long fluorescence lifetime; No photobleaching	Low extinction coefficient	[62] [63] [64] [65]
	MOFs	Improve detection sensitivity	Its potential toxicity to living tissue	[68]
	Laser materials	Improve optical properties	-	[69]
Single-walled carbon nanotubes	Nanocrystals SWCNTs	Improve optical properties High quantum yield, small background interference, good Light stability	-	[70] [78]

**Table 2:** Applications of near-infrared fluorescence immunoassay.

NIFP Types	NIFPs	Detection Object	Sample	Detection Limit	Reference
Near-infrared fluorescent material	Qijiachuan cyanine dye	BSA (bovine serum albumin) HAS (Human Serum Albumin) C-IgG	Serum Serum Serum	37 ng/mL 40 ng/mL 43 ng/mL	[26]
	FFOI	Human IgG	Human Serum	10 ng/mL	[81]
	Cy5 optical immunosensor	*Brucella* sp. antibody	Sick sheep serum	0.005–0.11 mg/mL	[89]
	MeCy5-OSu(VII)	Biopolyamine	Red blood cells of normal human	0.8–3.0 nmol/L	[30]
	SWCNTs	IgG	Serum	600 pmol/L	[78]
	NIR-antibody NIR-antibody NIR	Interleukin-6/C-reactive protein LP antigen LAM	Human sample Sample Sample to be tested	pg/mL 10 ng/mL 0.5 ng/mL	[83] [100] [101]
Near-infrared fluorescent quantum dot	quantum dot	chloramphenicol (CAP)	PBS	0.2 ng/mL	[57]
Near-infrared fluorescent rare earth complex	Lanthanides containing Eu ^3+^, Yb ^3+^ and Tm ^3+^	Chloramphenicol residue Stewart’s bacterial wilt of corn	Milk/PBS Corn seed	0.03–1 ng/mL 10^3^ cfu/mL	[94] [95]
	Lanthanides containing Eu^3+^/Yb ^3+^	Hepatitis B virus surface antigen and e antigen	Patient serum	0.0092 g·L^−1^/10 NCU·L^−1^	[97]

**Table 3:** Our team’s overviews of different pathogens near-infrared fluorescence immunoassay.

Pathogens	Label	Antibody	Detection Limit (CFU/mL)	Cross Reaction
*Salmonella*	Dylight800	*Salmonella* monoclonal/polyclonal antibody Goat anti-Rat IgG	0.5 × 10^3^	no
*Vibrio parahaemolyticus*	Dylight800	*Vibrio parahaemolyticus* monoclonal\polyclonal antibody Goat anti-Rat IgG	1.2 × 10^2^	no
*Vibrio cholerae*	Dylight800	Rat anti-*Vibrio cholerae* monoclonal\polyclonal antibodyGoat anti-Rat IgG	1.0 × 10^2^	no
*Listeria monocytogenes*	Dylight800	*Listeria monocytogenes* monoclonal\polyclonal antibody Goat anti-Rat IgG	5.0 × 10^2^	no

## Practical Applications

The applications of near-infrared fluorescence probes (NIFPs) in immunological analysis and clinically important biomarkers were also elaborated. Moreover, a NIFPs-based immunoassay adaptable for rapid detection of foodborne pathogens was also forecast. In 2017, our team developed an approach for detecting pathogens such as *Salmonella*, *Vibrio parahaemolyticus*, *Vibrio cholerae* and *Listeria monocytogenes* by near-infrared immunoassay.

## Abbreviations

NIFPs	near-infrared fluorescence probes
DOFLA	different-oriented fluorescence library approach
NIR	near-infared
ICG	goat anti human immunoglobulins
FFOI	fluorescent fiber-optic immunosenseor
gahg	goat anti human immunoglobulins
CEIA	Capillary electrophoresis-based immunoasssay
LFA	lateral flow assay
ICTSs	immunochromatographic test strips
NIFPs	near-infrared fluorescence probes
LP	*Legionella pneumophila*
SCWNTs	single-walled carbon nanotubes
HSA	Human Serum Albumin
CE-LIF	Capillary electrophoresis-laser-induced fluorescence
